# Epidemiology and clinical characteristics of biopsy-confirmed adult-onset IgA vasculitis in southern Sweden

**DOI:** 10.1136/rmdopen-2023-003822

**Published:** 2024-02-05

**Authors:** Michaela Thalen, Karl Gisslander, Mårten Segelmark, Jacob Sode, David Jayne, Aladdin J Mohammad

**Affiliations:** 1 Department of Clinical Sciences Lund, Rheumatology, Lund University, Lund, Sweden; 2 Department of Clinical Sciences Lund, Nephrology, Lund University, Lund, Sweden; 3 Department of Nephrology and Rheumatology, Skåne University Hospital, Lund, Sweden; 4 Department of Medicine, University of Cambridge, Cambridge, UK

**Keywords:** systemic vasculitis, epidemiology, incidence

## Abstract

**Objective:**

Immunoglobulin A vasculitis (IgAV) is the most prevalent primary childhood vasculitis in Sweden, but is considerably rarer in adults. This study aims to describe the epidemiology, clinical characteristics and renal outcome of adult-onset IgAV in Skåne, Sweden.

**Methods:**

The study area consisted of Skåne, the southernmost region of Sweden, with a population ≥18 years of 990 464 on 31 December 2010. Adult patients assigned the International Classification of Diseases-10 code for IgAV (D69.0) from 2000 through 2019 were retrospectively identified in a population-based database. Medical records were reviewed to validate the diagnosis of IgAV and extract data. Only patients with clinical manifestations of IgAV and biopsy-confirmed disease were included. The annual incidence and point prevalence of biopsy-confirmed IgAV were estimated.

**Results:**

Fifty-nine patients (19 women) were classified as having adult-onset IgAV. The incidence was 3 per 1 000 000 and was higher among men than women (4 vs 2/1 000 000, p=0.004). Ninety-seven per cent of patients presented with non-thrombocytopenic purpura, 78% with renal involvement, 59% with arthritis/arthralgia and 39% with gastrointestinal symptoms. Fifteen per cent developed chronic kidney disease stage ≥G3 a and one patient progressed to end-stage kidney disease during follow-up.

**Conclusion:**

Adult-onset IgAV is rare in southern Sweden with the incidence higher in men than in women. IgAV frequently affects the kidneys and leads to chronic kidney disease in adults, although the long-term renal outcome appears favourable compared with other small-vessel vasculitides affecting the kidneys.

**WHAT IS ALREADY KNOWN ON THIS TOPIC**

Adult-onset immunoglobulin A vasculitis (IgAV) is considered rarer than childhood-onset IgAV and often includes more severe organ involvement.

There are no approved therapies or management guidelines for adult-onset IgAV.

**WHAT THIS STUDY ADDS**

- We present the first epidemiological estimates of adult-onset IgAV in Sweden and describe the clinical characteristics, histopathology and treatments used in the population.
- IgAV frequently leads to chronic kidney disease in adults. End-stage kidney disease is less common than in other small-vessel vasculitides affecting the kidney.

**HOW THIS STUDY MIGHT AFFECT RESEARCH, PRACTICE OR POLICY**

- Further prospective multicentre studies may be conducted to establish diagnostic criteria and management guidelines for adult onset IgAV.
- Conducting organ biopsies with immunofluorescence for IgA in patients with clinically suspected IgAV will enhance diagnostic accuracy and provide a better understanding of the disease’s epidemiology.

## Introduction

Immunoglobulin A vasculitis (IgAV), formerly known as Henoch-Schönlein purpura, is a small vessel vasculitis displaying deposits of immunoglobulin A (IgA)–dominant immune complexes. Although the precise pathophysiological mechanism of IgAV is unknown, studies have shown that aberrantly glycosylated IgA_1_ plays a pivotal role.^1^ Common clinical presentations include non-thrombocytopenic purpura, arthritis/arthralgia, renal involvement and gastrointestinal disturbances.^2^

IgA vasculitis is the most prevalent primary childhood vasculitis in southern Sweden with an annual incidence of 175 per 1 000 000 population <18 years of age, with the highest proportion occurring in the first 10 years of life.^3^ The incidence and prevalence of IgAV among adults in Sweden have not been determined. Studies conducted in other regions indicate that adult-onset IgAV is less common than in children. In Spain and Slovenia, incidence rates have been estimated to range from 1.5 to 51 cases per 1 000 000 adults.^4 5^ It is worth noting that the reported incidence rate in Slovenia is much higher than that of other regions in Europe.^6–8^

In children, IgAV is mostly mild and self-limiting. Compared with childhood-onset disease, most studies of adult-onset IgAV report higher mortality, increased frequency of severe organ involvement and, especially, worse renal outcome.^5 9–13^ The disease in adults is more likely to follow a remission/relapsing course with an increasing burden of steroid and immune suppressive therapies.^14^ Renal involvement, potentially leading to chronic disease, is the most serious organ involvement in both children and adults.^5 9–13^

Most reports of IgAV thus far have focused on childhood-onset disease, and there are no approved therapies or management guidelines for adult-onset IgAV. Hence, there is a need to further investigate the clinical spectrum in adults. The epidemiology of IgAV varies among populations, and there is to date no estimated incidence or prevalence for adult-onset IgAV in Sweden. This study aims to (1) estimate the incidence and prevalence of adult-onset IgAV in southern Sweden, (2) describe clinical manifestations at onset and (3) assess the long-term renal outcome.

## Methods

### Study area and population

**Figure 1:**
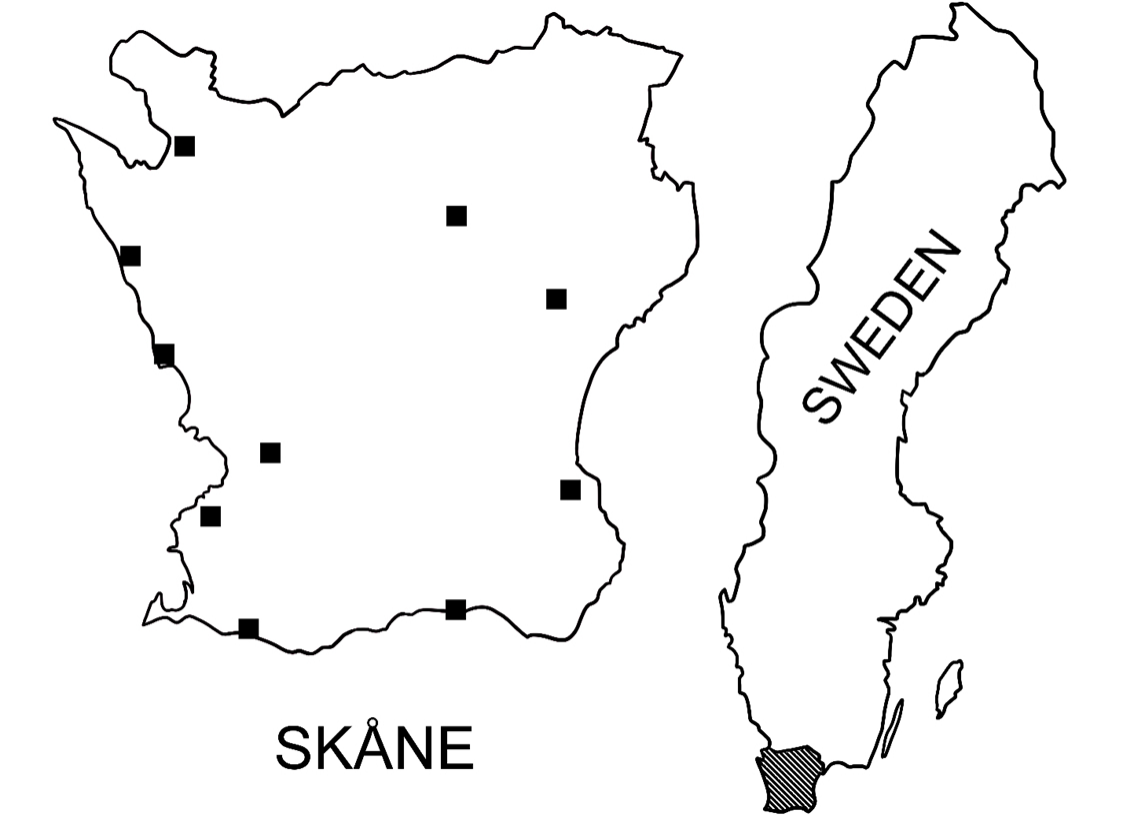
Map of Sweden and the study area (shaded). Hospitals in the study area are marked with a square.

The study was done in Skåne, the southernmost region of Sweden (11 303 km^2^, 2.5% of the total area of Sweden), which includes both highly urbanised and rural areas (figure 1). In December 2010, the population ≥18 years was 990 464 (50.7% women), ~10% of the total population of Sweden. Approximately 22% were foreign-born. The adult population increased by 195 081 from 2000 to the end of 2019.

The study area is served by 10 hospitals, all except one administered by Region Skåne, a public body with administrative and financial responsibility for providing healthcare to the inhabitants of Skåne. Individuals with suspected IgAV were generally referred by a family or emergency physician to the local department of dermatology, nephrology and/or rheumatology depending on predominant organ involvement. The department of pathology provides histology services to all physicians and hospitals in Skåne with a single computerised system. Patients from all hospitals in the study area were included.

### Patients

Patients ≥18 years who had been assigned the International Classification of Diseases (ICD)-10 code D69.0, allergic purpura, from 1 January 2000 through 31 December 2019 were identified retrospectively by search of the Skåne Healthcare Register (SHR). The SHR entries contain data of personal identification numbers, age, sex, place of residence, dates of clinical visits, hospital admission and discharge and healthcare providers, as well as up to eight diagnostic codes using the ICD-10 codes for every consultation. The register constitutes the basis for reimbursement and can be considered comprehensive.^15^

Cases of IgAV may be miscoded as IgA nephritis (IgAN) if non-renal features are mild and transient. To account for this possibility, we also searched the SHR for adult patients assigned ICD-10 code N03.8. There is no specific ICD code for IgAN, but, in Sweden, it is most commonly coded as N03.8, *chronic nephritic syndrome with other morphological changes*. Similarly, to rule out the possibility of missing miscoded patients with IgAV as cutaneous vasculitis, we conducted a search for the ICD-10 code L95.9, *vasculitis limited to the skin, unspecified*.

### Case ascertainment and classification

All cases identified by the initial search of the SHR were potentially eligible to be included in the study. The case records and histology reports were reviewed by three authors (MT, KG and JS) to confirm the diagnosis. Borderline cases were discussed with a fourth author (AJM) and the final judgement was based on consensus.

There are no validated classification criteria for adult-onset IgAV. According to the Chapel Hill Consensus Conference definitions (CHCC 2012), IgAV is vasculitis with IgA1-dominant immune deposits, affecting small vessels.^2^ In this study we included only biopsy-confirmed cases in patients with a diagnosis made at age 18 or older. Patients with hepatitis B or C were excluded to avoid misinterpretation of cryoglobulinaemic vasculitis.

### Definitions

*Biopsy-confirmed IgAV* was defined as leucocytoclastic vasculitis with IgA deposits in the vessel wall detected by direct immunofluorescence and/or a kidney biopsy with at least 1+ mesangial IgA deposit by direct immunofluorescence in combination with systemic symptoms compatible with IgAV.

*Baseline* was defined as onset of symptoms through 30 days post-diagnosis. Date of diagnosis was that of the first biopsy results.

*Haematuria* was defined as ≥10 red blood cells/high power field or a dipstick result of ≥2+.

*Proteinuria* was defined as an albumin-to-creatinine ratio of ≥3 g albumin/mol creatinine.

*Estimated glomerular filtration rate (eGFR*) was calculated by using the chronic kidney disease (CKD) epidemiology collaboration equation, and patients were considered to suffer from renal insufficiency if eGFR was <60 mL/min/1.73m^2^.^16^

*Increased IgA level* was defined as total IgA>4.5 g/L, and C reactive protein (CRP) was considered elevated at ≥3 mg/L based on the reference ranges used in Region Skåne hospitals.

*Fever* was body temperature >38°C.

*Obesity* was defined as body mass index >30 kg/m^2^.

*Recent infection* was defined as a history of bacterial or viral infection up to 3 months prior to onset of symptoms.

Relapse was defined as new clinical symptoms of IgAV requiring a new course of treatment or higher dosage of existing treatment.

CKD was classified according to the Kidney Disease Improving Global Outcomes criteria.^17^ Patients were considered to have CKD if there were documented findings of haematuria, proteinuria and/or eGFR<60 mL/min/1.73m^2^ in samples taken more than 3 months apart. The CKD stage was determined based on the last available serum creatinine.

### Data collection

General characteristics such as date of birth, sex, pre-existing comorbidities, date of disease onset and date of diagnosis were recorded. Baseline data of clinical manifestations of organ involvement, constitutional symptoms, laboratory data, pathology findings and treatment were collected. Vasculitis activity was quantified with Birmingham Vasculitis Activity Score.^18^ Baseline histological findings of skin vasculitis and IgA deposits on direct immunofluorescence were extracted from pathology reports. The renal biopsies were classified according to Pillebout *et al*.^19^

Patient records were followed from date of IgAV diagnosis to 31 December 2020, the date of the most recently available serum creatinine of those who left the region, date of first dialysis or renal transplant or death. Use of immunosuppressive agents and renin-angiotensin-aldosterone system (RAAS) blockers was recorded.

### Statistical analysis

Statistical analysis was performed using the Statistical Package for Social Sciences (IBM SPSS Statistics for Windows, V.27.0. Armonk, New York, USA: IBM Corp). To compare groups, Student’s t-test was conducted for normally distributed continuous variables and the Mann-Whitney U test for non-normally distributed continuous variables. Χ^2^ test was used to compare differences among categorical variables. When the expected count of categorical variables was fewer than five, Fisher’s exact test was used. P values≤0.05 were considered statistically significant. The 95% CI was calculated assuming a Poisson distribution of the observed cases. Continuous data were presented as mean values with SD or median values with IQR when appropriate.

For the incidence study, all newly diagnosed cases of adult-onset IgAV in the study area from 2000 through 2019 were included as the numerator. The denominator was the sum of the total adult population during the same period. For the prevalence estimates, the numerator was all patients with IgAV diagnosed after 2000 that were living in the study area on the prevalence estimate date (31 December 2019). The denominator was the adult population of the study area on that date.

## Results

**Figure 2:**
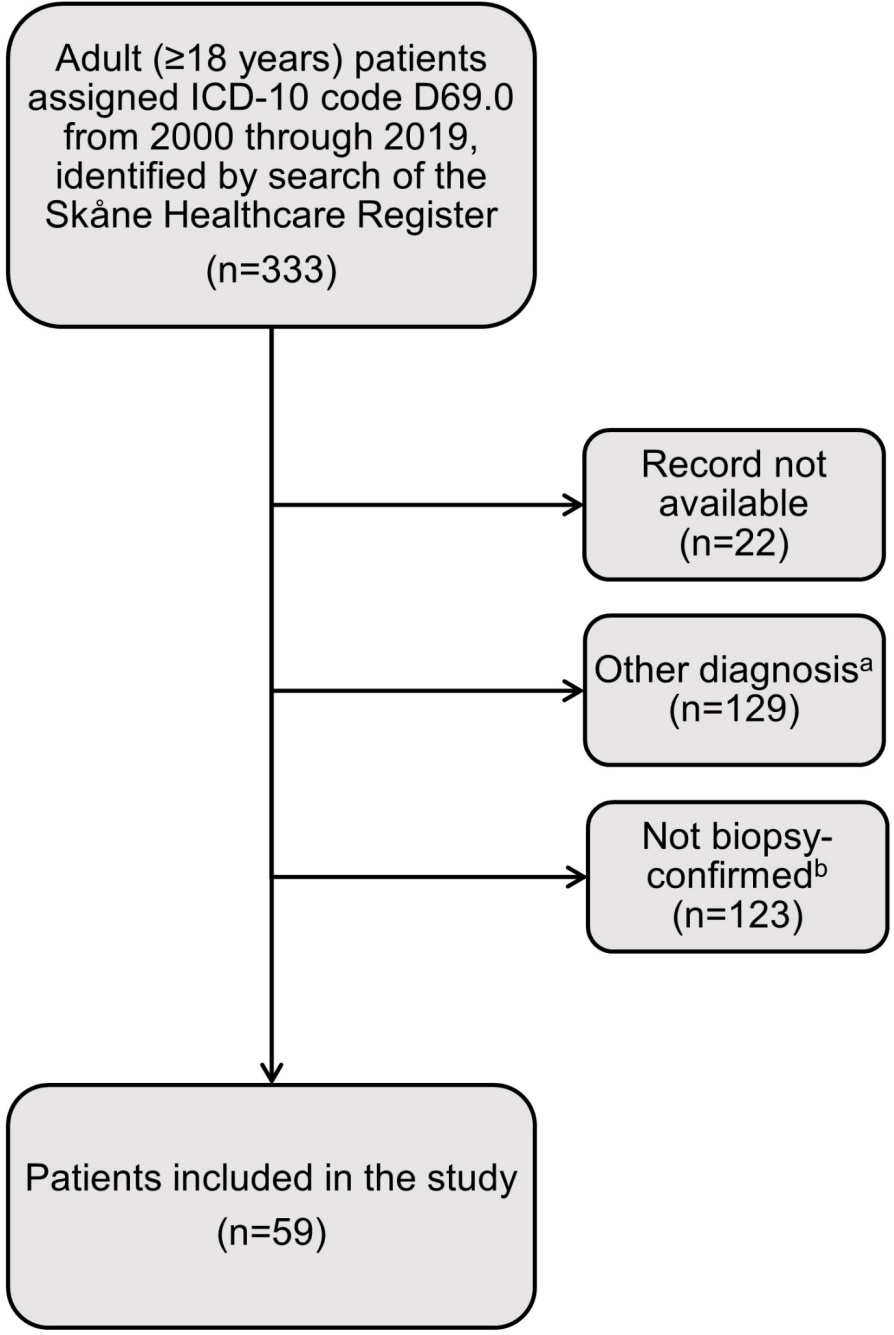
Flowchart of the study inclusion process. ^a^Includes patients with hepatitis (n=3), insufficient data, inaccurate use of the ICD-10 code (mostly allergic reactions) and changed diagnosis. ^b^Includes patients with possible immunoglobulin A vasculitis but no biopsy and/or immunofluorescence performed, or immunoglobulin A negative immunofluorescence. ICD, International Classification of Diseases.

**Figure 3:**
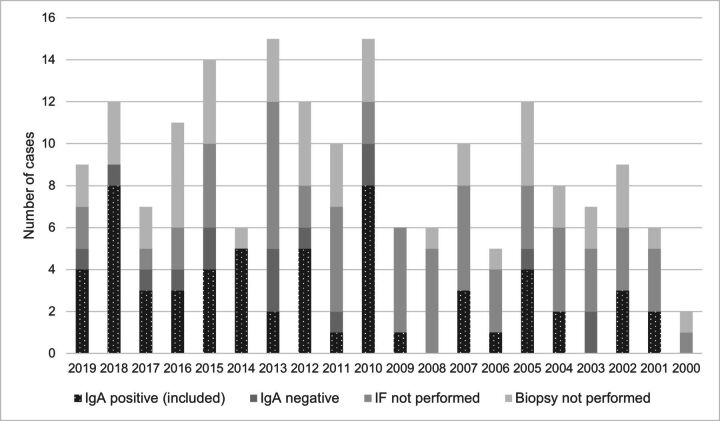
Numbers of the potential cases of IgA vasculitis (IgAV) identified based on the International Classification of Diseases-10 code D69.0, non-thrombocytopenic purpura and a clinical history compatible with IgAV that met inclusion and exclusion criteria for each year of the study period. IF, immunofluorescence; IgA, immunoglobulin A .

We identified 333 adults assigned ICD-10 code D69.0 for IgAV during the 2000−2019 study period. Of these, records were available for validation by case review for 311 (figure 2). One hundred and twenty-three individuals showing symptoms compatible with adult-onset IgAV with no biopsy, biopsy without immunofluorescence or negative immunofluorescence were excluded (figure 3). Fifty-nine (19 women) met all inclusion criteria and were included in this analysis.

The ICD-10 code N03.8 was assigned in 451 cases, of which we randomly sampled 113. Thirty-nine were diagnosed with IgAN 2000−2019, none of whom showed clinical manifestations or had a confirmed diagnosis of IgAV. The remaining N03.8 cases were patients with other kidney disease affecting glomeruli.

The search using ICD-code L95.9 identified 16 cases during the study period. One patient under 18 was excluded, and records for three cases were missing. One patient was already included under D69.0. Two patients (one woman) had skin biopsies showing vasculitis with positive IgA on immunofluorescence, despite no clinical suspicion for IgAV in the records. However, both cases presented with complicated clinical manifestations and were diagnosed with systemic lupus erythematosus and non-specific severe systemic illness and were consequently excluded from this study. Four patients showed symptoms compatible with IgAV but lacked positive biopsy or immunofluorescence. The remaining five patients had other diagnoses.

### Incidence

The annual incidence of adult-onset IgAV was estimated to be 3.0/1 000 000 adults (95% CI 2.2 to 3.8). The incidence was higher in men than in women: 4.0 (95% CI 2.8 to 5.3) versus 2.0 (95% CI 1.1 to 2.9); p=0.004. The highest age-specific incidence was estimated in the group 18–27 years, 5.7/1 000 000 (95% CI 3.0 to 8.3) (table 1).

**Table 1:** Incidence and point prevalence of biopsy-confirmed immunoglobulin A vasculitis per 1 000 000 adult (≥18 years) inhabitants of Skåne, Sweden, 2000−2019

	No. of cases	Incidence (95% CI)
All	59	3.0 (2.2 to 3.8)
Sex-specific incidence		
Male	40	4.0 (2.8 to 5.3)
Female	19	2.0 (1.1 to 2.9)
Age-specific incidence		
18–27	18	5.7 (3.0 to 8.3)
28–37	11	3.3 (1.4 to 5.3)
38–47	11	3.3 (1.4 to 5.3)
48–57	6	1.9 (0.4 to 3.4)
58–67	5	1.8 (0.2 to 3.3)
68–77	5	2.3 (0.3 to 4.4)
≥78	3	1.8 (0.0 to 3.9)

	No. of cases	Prevalence (95% CI)
All*	49	45.3 (32.6 to 58.0)
Sex-specific point prevalence		
Male	32	59.0 (38.6 to 79.4)
Female	17	35.3 (19.4 to 51.2)

*All patients living in the study area on the date of the point prevalence estimates (31 December 2019).

### Point prevalence

At the date of point prevalence estimates, 31 December 2019, 49 adults with IgAV were living in the study area (table 1). The prevalence of IgAV was estimated to be 45.3/1 000 000 adults (95% CI 32.6 to 58.0) and was higher in men: 59.0 (95% CI 38.6 to 79.4) versus 35.3 (95% CI 19.4 to 51.2); p=0.03. Table 2 compares the incidence and prevalence found in this study with results of other epidemiological studies of adult-onset IgAV across Europe.

**Table 2:** Epidemiological comparison of this study with selected studies from selected geographical European countries

	Sweden(this study)	Slovenia^5^	Spain^4^	UK^6^	Spain^7^	Denmark^8^
No. of adult patients	59	81	7	7 (CHCC)	27	NR
Study period	2000–2019	2010–2013	1994–2010	1990–1994	1988–1997	1977–1984
Biopsy-confirmed diagnosis	Yes	Yes	Yes	Yes	Yes	No
Definition of adult-onset (years)	≥18	≥18	≥15	>16	≥21	≥15
Region	Skåne	Ljubljana	Costa del Sol	Norfolk	Lugo	Copenhagen
Total study population	990 464	527 729	4 682 098	414 500	250 000	600 000
Incidence*	3.0	51	1.5	3.4	14	8
Prevalence*	45.3	NR	7.9	NR	NR	NR

*Per 1 000 000.

CHCC, Chapel Hill Consensus Conference definitions; NR, not reported.

### Demographics and clinical characteristics

The median age at diagnosis was 38 years (24–55): 40 (26–59) for men and 31 (19–51) for women (p=0.21) (table 3). The most common clinical presentation at onset was non-thrombocytopenic purpura (n=57, 97%). The two patients without purpura at onset presented with nephritis and developed purpura within 3 and 12 months.

**Table 3:** Baseline clinical data of men and women with adult-onset IgA vasculitis diagnosed in Skåne between 2000 and 2019

	All patients (n=59)	Men (n=40)	Women (n=19)	P value
Age at diagnosis, years, median (IQR)	38 (24–55)	40 (26–59)	31 (19–51)	0.21
Comorbidities, n (%)				
BMI>30	17 (29)	10 (25)	7 (37)	0.54
Hypertension	10 (17)	9 (23)	1 (5)	0.14
Diabetes mellitus	2 (3)	2 (5)	0	1.0
Recent infection, n (%)	19 (32)	12 (30)	7 (37)	0.56
Skin involvement, n (%)	57 (97)	38 (95)	19 (100)	1.0
Palpable purpura	57 (97)	38 (95)	19 (100)	1.0
Bullous/necrotic purpura	7 (12)	5 (13)	2 (11)	1.0
Joint involvement, n (%)	35 (59)	23 (58)	12 (63)	0.78
Arthritis	10 (17)	7 (18)	3 (16)	1.0
Arthralgia	32 (54)	21 (53)	11 (58)	0.78
Renal involvement, n (%)	46 (78)	33 (83)	13 (68)	0.31
Microscopic haematuria	40 (68)	28 (70)	12 (63)	0.75
Proteinuria	25 (42)	18 (45)	7 (37)	0.58
New onset renal insufficiency	9 (15)	7 (18)	2 (11)	0.31
Gastrointestinal involvement, n (%)	23 (39)	16 (40)	7 (37)	1.0
Abdominal pain	21 (36)	14 (35)	7 (37)	1.0
Bleeding	13 (22)	8 (20)	5 (26)	0.73
Nausea	6 (10)	4 (10)	2 (11)	1.0
Fever, n (%)	3 (5)	2 (5)	1 (5)	1.0
Laboratory findings at onset				
S-creatinine, μmol/L, median (IQR)	89 (71–102)	95 (84–119)	66 (61–73)	<0.0001*
eGFR, mL/min/1.73m^2^, median (IQR)	92 (74–109)	88 (60–102)	105 (83–124)	0.019*
CRP, mg/L, median (IQR)	15 (2–47)	16 (3–49)	11 (1–37)	0.25
IgA, g/L, mean (SD)	3.6 (1.5)	3.8 (1.7)	3.3 (1.1)	0.25
Albumin, g/L, mean (SD)	36 (7)	35 (7)	36 (7)	0.84
BVAS at diagnosis, mean (SD)	12 (6)	12 (6)	12 (5)	0.90
Initial treatment, n (%)				
Systemic GC	39 (66)	28 (70)	11 (58)	0.37
IS	3 (5)	3 (8)	0	0.54
ACEI/ARB	12 (20)	7 (18)	2 (26)	0.49

*P value<0.05.

ACEI/ARB, ACE inhibitor/angiotensin receptor blocker; BMI, body mass index; BVAS, Birmingham Vasculitis Activity Score; CRP, C reactive protein; eGFR, estimated glomerular filtration rate; GC, glucocorticosteroids; IgA, immunoglobulin A; IS, immunosuppressive drugs.

The second most common clinical presentation was renal involvement (n=46, 78%), primarily mild, consisting of microscopic haematuria (n=40) and non-nephrotic proteinuria (n=24). Gross haematuria was documented in two patients and nephrotic proteinuria in one. Haematuria was present in 87% of those with renal involvement. Among patients with proteinuria, the median albumin-to-creatinine ratio was 37.0 (12.5–77.5) g albumin/mol creatinine. Nine patients (15%) showed new onset renal insufficiency at baseline. Two elderly patients had renal insufficiency before onset of IgAV. No patient required renal replacement therapy at baseline.

Joint involvement was the third most common presentation (n=35, 59%), followed by gastrointestinal symptoms (n=23, 39%). One individual presented with mesenteric volvulus requiring laparotomy.

One patient developed peripheral polyneuropathy within the first month and another described headache at onset. One suffered from haemoptysis at onset.

### Laboratory results

The main laboratory findings are summarised in table 3. The eGFR (mL/min/1.73m^2^) was significantly lower in men (88 (60–102)) than in women (105 (83–124)); p=0.01. Serum IgA levels were elevated in 10 of 41 tested (24%). The median CRP level was 15 (2–47) mg/L and was elevated at baseline in 41 of 56 tested (73%).

### Histology

Baseline biopsy was conducted in included subjects: 36 skin biopsies, 10 renal biopsies and 13 combined skin and renal biopsies. One patient had a positive biopsy of the colon with confirmed vasculitis and IgA deposits in addition to skin and renal biopsies. Twenty-three (39%) underwent renal biopsy at baseline, 17 men and 6 women. Ten (17%) underwent a renal biopsy during the follow-up period. All kidney biopsies (n=33) showed mesangial IgA and C3 deposits.

Endocapillary proliferative glomerulonephritis was the most frequent finding (n=13), followed by mesangiopathic glomerulonephritis (n=5), focal and segmental glomerulonephritis (n=4) and endocapillary/extracapillary glomerulonephritis (n=2). One patient had a fibrotic kidney. The remaining biopsies (n=8) showed too few glomeruli to be classified.

### Treatment

Thirty-nine patients (66%) received oral glucocorticosteroids (GC) at diagnosis (table 3). The mean daily dose of initial systemic GC (as prednisolone-equivalent) was 40±19 mg/day. Three patients were treated with oral cyclophosphamide at diagnosis, 150 mg/day each. A 24-year-old man displaying severe systemic symptoms was treated with plasmapheresis.

Sixteen patients (27%) required immunosuppressive therapy during follow-up. Azathioprine was the most common treatment (n=15), followed by mycophenolate mofetil (n=4), methotrexate (n=3), rituximab (n=2) and cyclophosphamide (n=2). Adverse events led to discontinuation of azathioprine in six patients and rituximab in one.

Twelve patients (20%) received RAAS blockade at diagnosis. Fifteen patients (25%) were being treated with RAAS blockade at final follow-up.

### Outcome

Patients were followed for a median duration of 6.5 (IQR 2.8–11.8) years.

Twenty-one patients (36%) experienced relapsing disease during follow-up. Among them, 6 patients had one relapse, while the remaining 15 patients encountered three or more relapses.

At final follow-up, 27 (46%) had developed CKD: nine (33%) stage G1, nine (33%) stage G2, six (22%) stage G3a, one (4%) stage G3b and one (4%) stage G4. One female patient reached stage G5, developed end-stage kidney disease (ESKD) at age 35 and received a renal transplant 11.75 years after diagnosis. CKD was more prevalent among those who underwent renal biopsy at onset: 18 of 23 (78%) versus 9 of 36 (25%), p=0.0001.

Five patients died during the follow-up period, representing cumulative mortality of 8%. Causes of death included malignancy (n=2), stroke (n=1), complications following hip fracture (n=1) and unknown (n=1). The mean age at death was 83±10 years.

Four patients moved from the study area and were lost to follow-up.

## Discussion

In this population-based study, we present the first epidemiological estimates of adult-onset IgAV in Sweden. The incidence was comparable with that reported in other European studies, with the exception of a much lower incidence than reported in Slovenia. Although IgAV frequently causes CKD in adults, our study also showed that the renal prognosis is relatively good, with only 1 of 59 patients developing ESKD.

We previously reported an incidence of 175 per 1 000 000 for childhood-onset IgAV.^3^ Although inclusion criteria and disease definition differed, IgAV in children is almost 60 times that of our reported adult annual incidence in Skåne of 3 per 1 000 000. In childhood-onset disease, the highest proportion of cases occur within the first 10 years of life.^3^ The highest incidence in this study was found in the age group 18–27 years, in contrast to Hočevar *et al* 5 who reported incidence to increase with age.

Our incidence of adult-onset IgAV is at the lower end of the spectrum compared with estimates from other countries. It is within the range of rates reported in Spain, the UK and Denmark, but substantially lower than the incidence of 51/1 000 000 reported in Slovenia.^4–6 8^ The Slovenian study by Hočevar *et al* 5 used an extensive search strategy of obtaining records of IgAV-compatible biopsies from the Institute of Pathology (University of Ljubljana, Slovenia) in addition to searching for ICD-10 code D69.0, which might partially explain the discrepancy. Other factors such as interactions of genetic and environmental dynamics or diagnostic practices could also be involved. There are no validated criteria for adult-onset IgAV, and we used strict standards in this study. Since we excluded potential cases of IgAV with no available biopsy and those that lacked immunofluorescence, the true incidence is likely higher, as should also be the case for other studies based on a biopsy-confirmed diagnosis. Nevertheless, the healthcare register we used to identify patients is exhaustive, giving credence to our estimates.

With respect to other small vessel vasculitides, the incidence of adult-onset IgAV is lower than the incidence of anti-neutrophil cytoplasmic autoantibody (ANCA)-associated vasculitides in the same region of Sweden: 15.4 per 1 000 000 adults for granulomatosis with polyangiitis and 12.8 per 1 000 000 adults for microscopic polyangiitis.^20^ It is more common than oeosinophilic granulomatosis with polyangiitis (1.8/1 000 000),^20^ Behçet’s disease (2/1 000 000)^21^ and polyarteritis nodosa (0.9/1 000 000)^22^ in the same region.

Data of the prevalence of adult-onset IgAV are scarce, probably because IgAV is self-limiting in many cases, making it difficult to estimate. Tracy *et al* 23 reported a prevalence of 340–440 per 1 000 000 adult inhabitants based on clinical Read Codes, rather than using histology confirmed diagnosis. We would have attained similar results if we relied on the ICD-10 code only, but many patients had other diagnoses or insufficient data to confirm IgAV and were excluded.

Male predominance has been a frequent finding in studies of both childhood-onset and adult-onset IgAV.^11 13 24^ Our study similarly showed higher incidence and point prevalence in men. The basis of the difference is not known.

In general, the baseline clinical characteristics in this study corresponded with previously published studies.^10 11 13 24^ Overall, the disease pattern does not appear to show sex differences, with the significant exception of lower eGFR at onset in men, suggesting more severe initial renal involvement. This would be interesting, since women are often more severely affected by other small vessel vasculitides with renal involvement and have previously been shown to have poorer IgAV-related renal outcome.^25 26^ The only patient in our study to reach ESKD was female.

Two of our patients presented with nephritis, initially diagnosed as IgA nephropathy, months before the characteristic rash appeared. While it is known that gastrointestinal and joint involvement might precede purpura by several weeks, it is considered rare for nephritis to be the first clinical symptom. Yet, a Japanese retrospective study by Kamei *et al* 27 showed that 11% of children with IgAN developed purpura post-diagnosis. There is to date no consensus on disease classification in such cases. In this study, we decided to reclassify two patients from IgAN to IgAV, as the time between nephritis and purpura was less than a year in both cases. This decision was made in accordance with the CHCC 2012 definitions.^2^ It is also a subjective judgement, as the debate on the relationship between IgAN and IgAV is ongoing.

CKD was frequent in our patient population. The rate of CKD stages G1 and G2 is likely higher than we calculated, since follow-up data regarding haematuria and proteinuria were sparse for patients with mild symptoms. However, only 1 (2%) patient in our study received renal replacement therapy during follow-up. In contrast, Pillebout *et al* 19 reported in 2002 that 27 (11%) of their 250 patients with adult-onset IgAV-associated nephritis reached ESKD, almost half of those within 3 years. Similarly, Coppo *et al* 26 observed that 13% of patients with adult IgAV with renal involvement required dialysis after a mean follow-up of 5.5 years. A feasible explanation for this difference is that all patients in these studies had renal disease confirmed by kidney biopsy. More recent studies of patients with baseline characteristics similar to ours have reported a low rate of ESKD as well, suggesting that the renal outcome might be better than previously thought.^13 14 24^

The long-term renal outcome also seems to be favourable compared with other small vessel vasculitides affecting the kidneys. For comparison, kidney survival at 1 year after biopsy-confirmed anti-glomerular basement membrane disease may be as low as 13.5%.^28^ A 30-year follow-up study of ANCA-associated glomerulonephritis reported an estimated 5-year renal survival of only 54%, not death-censored and as many as 14% of patients were on dialysis at time of the renal biopsy.^29^ The same study also showed an improvement over the years, and suggested earlier diagnosis and better treatment as possible explanations. This might also be the case for adult-onset IgAV.

The findings of this study must be interpreted with consideration of its limitations. First, selecting only biopsy-confirmed cases might exclude patients with benign or typical presentation and result in an underestimate of incidence. Moreover, cases that resolve naturally without being assigned an ICD-10 code should also be considered. Second, IgAV can undergo long-term remission or be cured, which complicates calculating prevalence. We acknowledge that our prevalence figures are based on individuals who were previously diagnosed with biopsy-confirmed IgAV, without taking into consideration whether they are currently on active treatment.

There are also several strengths. First, all cases were biopsy-confirmed, hence no misdiagnoses. Second, the comparison of sexes gives further insight into the disease spectrum. Lastly, few studies have estimated the incidence and prevalence of adult-onset IgAV. To the best of our knowledge, this study is the first of its kind in Sweden and is the first study to describe incidence and prevalence of adult-onset IgAV in a Nordic country since 1988. We have extensively studied the epidemiology of various vasculitides in southern Sweden, and this study enables comparisons with other vasculitides in the same area as well as generates unique epidemiological insights that can potentially be applied to similar populations across northern and western Europe.

## Data Availability

No data are available. All data relevant to the study are included in the article or uploaded as supplementary information.
